# Role of K^+^ and Ca^2+^ Channels in the Vasodilator Effects of *Plectranthus barbatus* (Brazilian Boldo) in Hypertensive Rats

**DOI:** 10.1155/2023/9948707

**Published:** 2023-11-17

**Authors:** Jeniffer Cristóvão Moser, Rita de Cássia Vilhena da Silva, Philipe Costa, Luisa Mota da Silva, Nadla Soares Cassemiro, Arquimedes Gasparotto Junior, Denise Brentan Silva, Priscila de Souza

**Affiliations:** ^1^Postgraduate Program in Pharmaceutical Sciences, Nucleus of Chemical-Pharmaceutical Investigations, University of Vale do Itajaí, Itajaí, Brazil; ^2^Laboratory of Natural Products and Mass Spectrometry, Faculty of Pharmaceutical Sciences, Food and Nutrition, Federal University of Mato Grosso do Sul, Campo Grande, Mato Grosso do Sul, Brazil; ^3^Laboratory of Cardiovascular Pharmacology, Faculty of Health Sciences, Federal University of Grande Dourados, Dourados, Mato Grosso do Sul, Brazil

## Abstract

*Plectranthus barbatus*, popularly known as Brazilian boldo, is used in Brazilian folk medicine to treat cardiovascular disorders including hypertension. This study investigated the chemical profile by UFLC-DAD-MS and the relaxant effect by using an isolated organ bath of the hydroethanolic extract of *P. barbatus* (HEPB) leaves on the aorta of spontaneously hypertensive rats (SHR). A total of nineteen compounds were annotated from HEPB, and the main metabolite classes found were flavonoids, diterpenoids, cinnamic acid derivatives, and organic acids. The HEPB promoted an endothelium-dependent vasodilator effect (~100%; EC50 ~347.10 *μ*g/mL). Incubation of L-NAME (a nonselective nitric oxide synthase inhibitor; EC50 ~417.20 *μ*g/mL), ODQ (a selective inhibitor of the soluble guanylate cyclase enzyme; EC50 ~426.00 *μ*g/mL), propranolol (a nonselective *α*-adrenergic receptor antagonist; EC50 ~448.90 *μ*g/mL), or indomethacin (a nonselective cyclooxygenase enzyme inhibitor; EC50 ~398.70 *μ*g/mL) could not significantly affect the relaxation evoked by HEPB. However, in the presence of atropine (a nonselective muscarinic receptor antagonist), there was a slight reduction in its vasorelaxant effect (EC50 ~476.40 *μ*g/mL). The addition of tetraethylammonium (a blocker of Ca^2+^-activated K^+^ channels; EC50 ~611.60 *μ*g/mL) or 4-aminopyridine (a voltage-dependent K^+^ channel blocker; EC50 ~380.50 *μ*g/mL) significantly reduced the relaxation effect of the extract without the interference of glibenclamide (an ATP-sensitive K^+^ channel blocker; EC50 ~344.60 *μ*g/mL) or barium chloride (an influx rectifying K^+^ channel blocker; EC50 ~360.80 *μ*g/mL). The extract inhibited the contractile response against phenylephrine, CaCl_2_, KCl, or caffeine, similar to the results obtained with nifedipine (voltage-dependent calcium channel blocker). Together, the HEPB showed a vasorelaxant effect on the thoracic aorta of SHR, exclusively dependent on the endothelium with the participation of muscarinic receptors and K^+^ and Ca^2+^ channels.

## 1. Introduction

Hypertension is a multifactorial chronic disease associated with kidney, heart, and brain complications. It increases the risk of cardiovascular events and is responsible for the increase in morbidity and mortality worldwide [1], leading to about ten million deaths per year [2].

Despite its positive impacts, the antihypertensive medication is also known for its side effects, such as dizziness, tiredness, depression, insomnia, impotence, and migraines [3], which leads to lower adherence to treatment, opening space for herbal medicine. The use of plant species for the treatment of various diseases has been taking place for thousands of years. It is present in several cultures around the world [4], and after a period when the focus of the pharmaceutical industry was on new chemical technologies, interest in natural product inputs was rescued due to the vast possibility of identifying new compounds and developing new medicines that nature can provide [5].

*Plectranthus barbatus* Andrews (Lamiaceae), known as Brazilian boldo or false boldo, is popularly used by the Brazilian population for several health purposes. Traditionally, different preparations obtained from its leaves are indicated for the treatment of gastrointestinal symptoms, such as diarrhea, stomachaches, and indigestion. Furthermore, it can also be used for respiratory disorders, inflammatory processes, certain central nervous system disorders, and the treatment of hypertension [6–8].

In the review by Alasbahi and Melzig [9], the authors gathered some studies on the cardiovascular and vasorelaxant effects associated with *P. barbatus*. These studies were carried out with a well-known compound from the plant, the labdane diterpenoid forskolin. This was isolated in 1977, initially called coleonol; it was found that this compound is identified only in the roots of *P. barbatus*. Scientific records indicate that this compound increased heart rate and decreased blood pressure in an experimental model of rats, dogs, cats, and spontaneously hypertensive rats (SHR). These pharmacological effects are associated with the activation mechanism of adenylyl cyclase, which in turn increases cyclic adenosine monophosphate.

As there is no preclinical or clinical evidence in the literature of the effects of this plant on the systems that contribute to the control of blood pressure, according to Brazilian popular use, the objective of this present study was to evaluate the effect and the mechanisms involved in the relaxation induced by the hydroethanolic extract obtained from the leaves of *P. barbatus* in isolated rat aorta model, in addition to evaluating the phytochemical composition of this extract.

## 2. Materials and Methods

### 2.1. Vegetal Material and the Extract Preparation

The leaves of *P. barbatus* (850 g) were collected in the city of Joinville, Brazil, in March 2018. A voucher specimen was deposited in the Joinville Herbarium of the University of the Region of Joinville under number 3974.

After drying and powdering, the leaves were subjected to maceration with ethanol and water 7 : 3 for 7 days. The extract was concentrated by a rotary evaporator and lyophilized to obtain the hydroethanolic extract of *P. barbatus* (HEPB) with a 6.5% yield. Then, it was held in a freezer (-20°C) until use.

### 2.2. UFLC-DAD-MS Analysis

An equipment Shimadzu LC-20AD UFLC chromatograph coupled to a diode array detector and a mass spectrometer micrOTOF III (Bruker Daltonics) in line was used to analyze the chemical profile from the HEPB. The samples were analyzed in both negative and positive ion modes. Nitrogen was applied as dry (9 L/min), nebulizer (4 bar), and collision gas. Chromatographic analyses were performed on a Kinetex C18 column (2.6 *μ*m, 150 × 2.1 mm, Phenomenex), which was maintained at 50°C and a flow rate of 0.3 mL/min. Acetonitrile (B) and ultrapure water (A) with 0.1% formic acid (*v*/*v*) were used as the mobile phase. The gradient elution was 0-2 min, 3% B; 2-25 min, 3-25%; 25-40 min, 25-80%; and 40-43 min, 80% B, followed by a 5 min column washing and reconditioning. The extracted sample was prepared at 5 mg/mL in methanol and ultrapure water (7 : 3, *v*/*v*), filtered by syringe filters (Millex®, PTFE, 0.22 *μ*m), and injected 3 *μ*L into the chromatographic system via autosampler. The annotation of the compounds was performed by the spectral UV, mass spectra data, and fragmentation profile compared to the literature.

### 2.3. Animals

Male Wistar normotensive rats (NTR) and spontaneous hypertensive rats (SHR) at three to four months of age were maintained at controlled room temperature (22 ± 2°C), in a light-dark cycle of 12/12 hours, with free access to water and food. They were provided by the Central Bioterium of Universidade do Vale do Itajaí (UNIVALI). The methodologies and procedures followed the experimental protocols previously approved by the Ethics Committee for the Use of Animals of the UNIVALI (no. 053/18 p). All the research was conducted in accordance with the internationally accepted principles for laboratory animal use and care.

### 2.4. Rat-Isolated Aorta Model

To remove the thoracic aorta, the animals from both groups, NTR and SHR, were anesthetized intraperitoneally with ketamine (80 mg/kg) and xylazine (10 mg/kg). The descending thoracic aorta artery was removed and transferred to a recipient containing physiological saline solution (PSS; composition in mM: NaCl 110.8, KCl 5.9, NaHCO_3_ 25, MgSO_4_ 1.07, CaCl_2_ 2.49, KH_2_PO_4_ 2.33, and glucose 11.51) heated to 37°C to remove connective tissue. Then, the vessel was sectioned into rings measuring approximately five mm in length. The obtained aortic rings were attached to two metallic rods conditioned in organ baths (with the capacity of 2 mL) containing the PSS and constantly aerated with 95% O_2_ and 5% CO_2_, kept at a temperature of 37°C, and submitted to a basal tension of 1 g. The isometric contraction was recorded through a signal amplifier and connected to a computer containing specific integration software (WinDaq Software, DATAQ Instruments, Akron, Ohio, USA). After 60 min of tissue stabilization, with PSS changes at 15 min, the preparations were contracted with a potassium chloride solution (60 mM, KCl) to identify tissue responsiveness. After a new interval of 30 min for stabilization of the preparations, a contraction was induced by the addition of phenylephrine (Phe, 1 *μ*M), followed by the administration of acetylcholine (Ach, 1 *μ*M) in the tonic phase of the contraction. Vessels with functional endothelium were considered for those preparations that obtained a relaxation equal to or greater than 80%.

### 2.5. Experimental Protocols

The pathways and mechanisms explored in this study followed the protocols and drug concentrations previously described in the literature [10–12].

#### 2.5.1. Effect of *P. barbatus* on Vascular Reactivity

After verifying the integrity of the endothelium, the rings were washed three consecutive times with PSS for a further stabilization period of 60 min. To investigate if the HEPB induces relaxation on aorta rings, the preparations were precontracted with Phe (1 *μ*M), and on the tonic phase of contraction, cumulative concentrations of the HEPB (0.3 to 1000 *μ*g/mL) were added to the bath. After this initial screening, all the following experiments were performed with SHR and aortic rings with functional endothelium.

#### 2.5.2. Assessment of the Role of Membrane Receptors and Endothelial Mediators on HEPB-Induced Relaxation

After the tissue preparation and aortic viability verification described above, to evaluate the involvement of muscarinic (M_3_) and *β*-adrenergic receptors, prostacyclin, nitric oxide, and the enzyme guanylate cyclase, different preparations were incubated in the organ bath for 30 min with atropine (Atro, 1 *μ*M, a nonselective muscarinic receptor antagonist), or propranolol (Prop, 1 *μ*M, a nonselective *α*-adrenergic receptor antagonist), or indomethacin (Indo, 10 *μ*M, a nonselective cyclooxygenase enzyme inhibitor), N*ω*-nitro-L-arginine methyl ester hydrochloride (L-NAME, 100 *μ*M, a nonselective nitric oxide synthase inhibitor), or 1H-[1,2,4]oxadiazolo[4,3-a]quinoxalin-1-one (ODQ, 10 *μ*M, a selective inhibitor of the soluble guanylate cyclase enzyme). In the presence of these substances (one in each preparation), a new contraction was induced by Phe, and, in the tonic phase of this contracting, the HEPB was added in cumulative concentrations of 0.3-1000 *μ*g/mL.

#### 2.5.3. Evaluation of the Role of K^+^ Channels in the Vascular Effects of HEPB

After stabilization, different preparations of the aortic rings were incubated with the following K^+^ channel blockers: tetraethylammonium (TEA, 10 or 1 mM), a nonselective K^+^ channel blocker, or glibenclamide (GLI, 10 *μ*M), an ATP-sensitive K^+^ channel blocker, or 4-aminopyridine (4-AP, 1 mM), a voltage-gated K^+^ channel blocker, or barium chloride (BaCl_2_, 10 *μ*M), an influx rectifying K^+^ channel blocker. In the presence of these substances (one in each preparation), a new contraction was induced by Phe, and, in the tonic phase of this contraction, the HEPB was added in cumulative concentrations of 0.3-1000 *μ*g/mL.

#### 2.5.4. Investigation of the Role of Extra- and Intracellular Ca^2+^ Channels in the Vascular Effects of HEPB

After stabilization, to evaluate the involvement of the extracellular Ca^2+^ channels, the PSS was replaced by a calcium-free depolarizing PSS (KCl, 60 mM). The preparations were kept in this solution for 30 min for stabilization and then incubated with 100, 300, and 1000 *μ*g/mL of HEPB for 30 min. In the presence of HEPB, cumulative concentration-response curves were built with calcium chloride solution (CaCl_2_, 10 *μ*M-100 mM). As a control, a preparation without the addition of the extract was used, and as a positive control, a preparation with the addition of the voltage-dependent calcium channel blocker nifedipine (NIFE, 1 *μ*M) was used.

To assess the involvement of intracellular calcium in the vasorelaxant effect of the extract, after confirming the presence of the endothelium, for another 30 min, the preparations were washed several times with calcium-free PSS. After the stabilization, the aortic rings were exposed to different extract concentrations (100, 300, and 1000 *μ*g/mL). After 30 minutes in the presence of the HEPB, a new contraction was induced by Phe (1 *μ*M), KCl (60 mM), or caffeine (100 mM). As a control, preparations were made without adding the extract and others with NIFE (1 *μ*M).

### 2.6. Statistical Analysis

Results were expressed as the mean ± standard error of the mean (*n* = 6‐8 animals in each group). One- or two-way analysis of variance (ANOVA) was used for the statistical analysis, followed by the Bonferroni test. A *p* value less than 0.05 was considered statistically significant.

## 3. Results and Discussion

**Figure 1:**
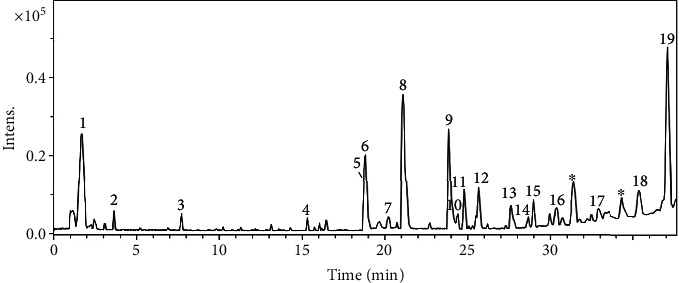
Base peak chromatogram obtained in positive ion mode from hydroethanolic extract of *P. barbatus* (HEPB).

The HEPB was analyzed by UFLC-DAD-MS, and its constituents were annotated. A total of 19 compounds were annotated (Figure 1 and Table 1) based on ultraviolet (UV), MS, and MS/MS spectral data compared with data in the literature. The main metabolite classes were flavonoids, diterpenoids, cinnamic acid derivatives, and organic acids.

Peak 1 showed no UV absorption and exhibited an intense ion at *m/z* 191.0202 [M-H]^−^ (C_6_H_7_O_7_^−^), which was putatively identified as citric acid. Compound 2 (*m/z* 197.0461 [M-H]^−^, C_9_H_9_O_5_^−^) showed a UV spectrum similar to a galloyl chromophore (*λ*_max_ ≈ 281 nm), and its fragmentation pattern indicated the losses of water (18 *u*) and CO molecules (28 *u*), which is compatible to syringic acid [13]. The metabolites 4 and 8/11 revealed absorption bands at 299/313 and 299/325 nm, suggesting chromophores of coumaroyl and caffeoyl groups. The peak 4 (*m/z* 279.0529 [M-H]^−^, C_13_H_11_O_7_^−^) yielded the fragment ion at *m/z* 163 relative to the coumaric acid. Compound 8 exhibited an intense ion at *m/z* 359.0752 [M-H]^−^, compatible with molecular formula C_18_H_16_O_8_. Additionally, the fragment ions at *m/z* 197 and 179 are relative to salvianic acid A and caffeic acid, respectively, yielded from losses 162 and 180 *u.* Peak 11 showed ion at *m/z* 207.0671 [M-H]^−^ and the fragment ion at *m/z* 161 [M-H]^−^ yielded from subsequent losses of H_2_O (18 *u*) and CO molecules (28 *u*). Thus, 4, 8, and 11 were annotated as *p*-coumaroyl malate, rosmarinic acid, and di-*O*-methyl caffeic acid, respectively [14, 15].

The compounds 5, 6, and 9 revealed two intense absorption bands at ≈268 and 348 nm wavelengths on UV spectra, indicating flavonols. Compounds 5 and 9 presented the molecular formulas C_22_H_22_O_12_ (*m/z* 477.1053 [M-H]^−^) and C_16_H_12_O_7_ (*m/z* 315.0521 [M-H]^−^), respectively. From metabolite 5, the loss of 162 *u* confirmed the *O*-hexosyl substituent, besides the subsequent loss of 15 *u* (CH_3_^•^) suggested *O*-methyl substituent on the aglycone quercetin. In compound 6, the loss of 176 *u* confirmed the presence of the *O*-glucuronyl substituent. Therefore, the compounds 5, 6, and 9 were identified as O-hexosyl *O*-methyl quercetin, *O*-glucuronyl kaempferol, and *O*-methyl quercetin [16, 17].

The metabolites 12 and 14-15 showed UV spectra similar to flavones, with two absorption bands at ≈275 and 336 nm. The compound 14 (*m/z* 475.0884 [M-H]^−^, C_22_H_19_O_12_^−^) presented the fragment ions at *m/z* 299 and 284 [M-H]^−^, which are yielded from subsequent losses of a glucuronyl (176 *u*) and a methyl groups (15 *u*). The metabolites 12 and 15 exhibited intense ions at *m/z* 285.0410 and 269.0450 [M-H]^−^, respectively. Thus, 12 and 14-15 were identified as luteolin, *O*-glucuronyl *O*-methyl scutellarein, and apigenin, respectively [14–18].

The metabolite 13 did not show absorption on UV and displayed a deprotonated ion at *m/z* 463.1985, while the metabolite 17 revealed an absorption band at the wavelength 280 and molecular formula C_22_H_28_O_8_ determined from the ion *m/z* 419.1716 [M-H]^−^. They were annotated as the diterpenoids cyclobutatusin (13) and coleon H (17), which are commonly described from *Plectranthus barbatus*, and their spectral data were compatible with them [9–19].

**Figure 2:**
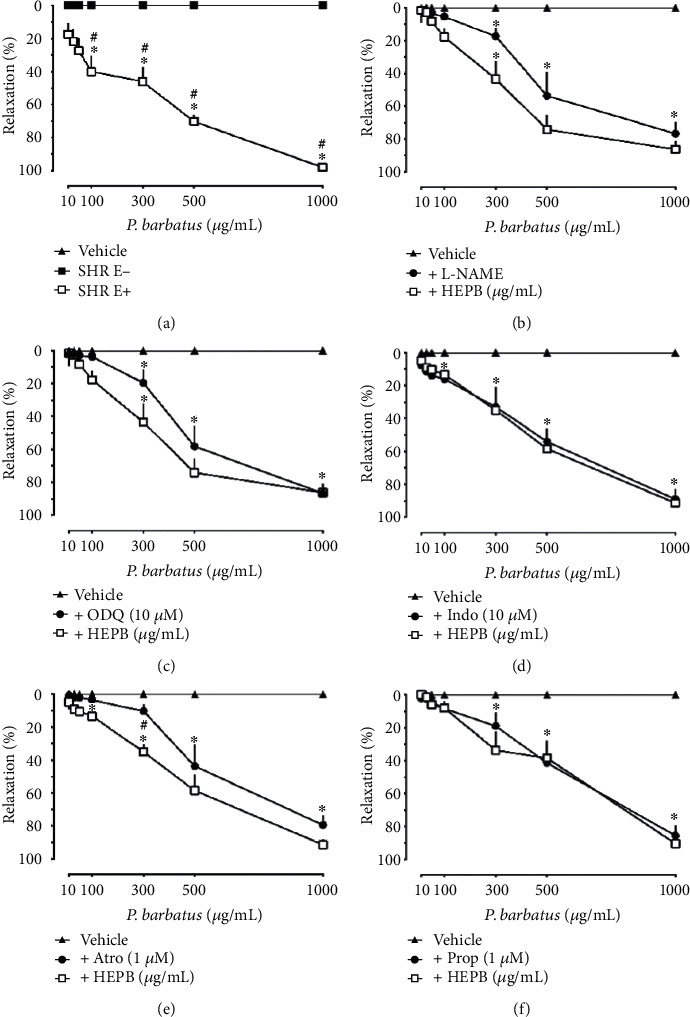
Endothelium-dependent relaxation of hydroethanolic extract of *P. barbatus* (HEPB) on aortic rings of SHR. Cumulative concentrations of HEPB were added to the Phe-contracted aortic rings. E+ and E- indicate endothelium-intact and endothelium-denuded preparations, respectively (a). Effect of HEPB in the presence of L-NAME (b), ODQ (c), Indo (d), Atro (e), and Prop (f). ^∗^*p* < 0.05 when compared to the vehicle group and ^#^*p* < 0.05 when compared to the HEPB group.

Initially, our objective was to evaluate whether the extract promoted vascular relaxation in both NTR and SHR. In increasing cumulative concentrations, HEPB (0.3-1000 *μ*g/mL), no relaxing effect on aortic rings lacking functional endothelium was observed, while it promoted vasorelaxant action in endothelium-preserved aortic rings, previously contracted with Phe, in both NTR (around 40%; data not shown) and SHR (around 100%; Figure 2(a)), EC50 values of ~449.00 *μ*g/mL and ~347.10 *μ*g/mL, respectively. The use of SHR for a preclinical investigation related to cardiovascular disease is considered the gold standard due to its similarity in the development of high blood pressure with essential hypertension developed by humans, as well as the progression of the disease to cardiac and renal complications [20], thus reinforcing the relevance of the significant data obtained with the aortic rings of SHR.

Hypertension and cardiovascular diseases arise because of changes in the cardiovascular system, especially in the vessels, where the endothelium is compromised and, consequently, the functioning of several mechanisms is dependent on the preservation of this vascular tissue [21]. We observed that the ability of the extract to modulate vascular tone occurred significantly in the aortic rings of SHR with the presence of functional endothelium. To assess the mechanism involved in vasorelaxation, further investigations were carried out only in SHR. Considering that the HEPB-induced relaxation is entirely dependent on the endothelium and that nitric oxide (NO) is the main endothelium-derived relaxation factor, the following data to be presented focus on this pathway. NO is considered one of the main mediators of cellular processes; it is produced in endothelial cells from L-arginine, having an important vasorelaxant action. It diffuses into the smooth muscle cell and interacts with the enzyme-soluble guanylate cyclase (sGC), making it active, which in turn results in the formation of cyclic guanosine monophosphate (cGMP), causing vascular smooth muscle cell relaxation [22]. The previous addition of L-NAME (100 *μ*M), a nonselective inhibitor of the enzyme nitric oxide synthase (NOS), as well as the prior addition of ODQ (10 *μ*M), an inhibitor of the sGC enzyme, in sufficient concentrations to prevent the relaxation induced by Ach, was unable to significantly interfere with the relaxation observed by adding HEPB in aortic rings previously contracted by Phe (Figures 2(b) and 2(c)), EC50 values of ~417.20 *μ*g/mL and ~426.00 *μ*g/mL, respectively. The results presented here suggest that the NO/sGC/cGMP pathway does not seem to be essential for the vascular effects of HEPB.

When Indo (Figure 2(d)) or Prop (Figure 2(f)) were incubated, the vasorelaxation provided by adding the HEPB concentrations was not modified, EC50 values of ~398.70 *μ*g/mL and ~448.90 *μ*g/mL, respectively. Indo is a nonsteroidal anti-inflammatory drug (NSAID), a classic nonselective inhibitor of the cyclooxygenase enzyme, which generates a series of prostanoids from arachidonic acid [23]. Prop is a drug that acts nonselectively by blocking *β*-adrenergic receptors competitively in vascular muscle cells but also, for information, in bronchial cells and the myocardium [24]. According to the data presented, we can suggest that the vasorelaxant effect of the HEPB does not depend on the generation of prostanoids, as it does not seem to involve the activation of *β*-adrenergic receptors. On the other hand, in the presence of Atro, a nonselective muscarinic receptor antagonist (Figure 2(e)), there was a slight but statistically significant reduction in the relaxation potential of the extract at a concentration of 300 *μ*g/mL, EC50 value of ~476.40 *μ*g/mL, while maximal relaxation was unaltered, suggesting that at least a small part of the vasorelaxant effect evoked by the extract seems to depend on the activation of this pathway.

Studies show that endothelium-derived factors, such as NO and PGI_2_, produce vasodilation by activating K^+^ channels in the cell membrane. The role of K^+^ channels is related to the regulation of membrane potential through the efflux of K^+^ from the cell and membrane hyperpolarization that leads to the closing of voltage-gated Ca^2+^ channels and consequent smooth muscle relaxation [25]. K^+^ channels are protein structures present in several types of cells; they function as pores in the membranes that allow the passage of K^+^; however, these channels differ according to their functional properties, and in vascular smooth muscle, they have already been identified as voltage-gated K^+^ channels (K_V_), ATP-sensitive K^+^ channels (K_ATP_), inward-rectifying K^+^ channels (K_IR_), and Ca^2+^-activated K^+^ channels (K_Ca_) [26].

**Figure 3:**
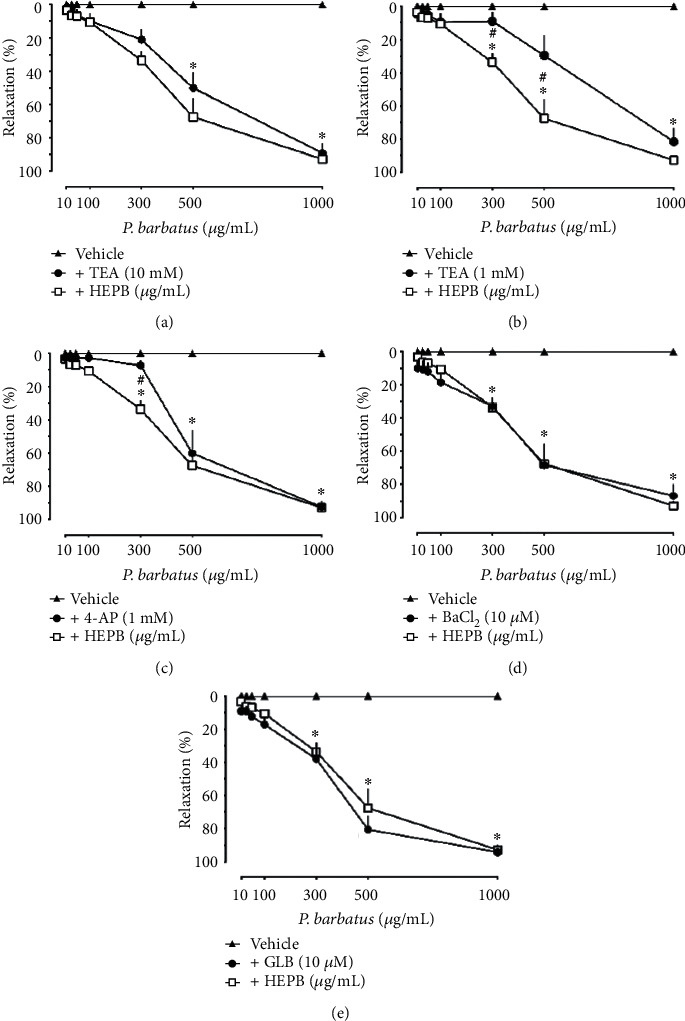
Role of K^+^ channels in the relaxant effect induced by the hydroethanolic extract of *P. barbatus* (HEPB). Cumulative concentrations of HEPB were added to the Phe-contracted aortic rings. Effect of HEPB in the presence of TEA 10 mM (a), TEA 1 mM (b), 4-AP (c), BaCl_2_ (d), and GLB (e). ^∗^*p* < 0.05 when compared to the vehicle group and ^#^*p* < 0.05 when compared to the HEPB group.

When investigating the involvement of K^+^ channels, we proceeded with the incubation of TEA in the concentration of 10 mM (Figure 3(a)) and 1 mM (Figure 3(b)), which acts as a nonselective blocker of K^+^ channels and a blocker of K_Ca_, respectively. TEA, in the concentration of 1 mM (EC50 value of ~611.60 *μ*g/mL), but not with 10 mM (EC50 value of ~462.90 *μ*g/mL), was able to significantly reduce the relaxing effect of the extract, suggesting the involvement of K_Ca_ channel type in the vascular effects induced by HEPB.  Figure 3(c) demonstrates the vasorelaxant effect of the HEPB in the presence of 4-AP, which acts by blocking K_V_, where a discrete but significant reduction in the relaxing effect of the extract (EC50 value of ~380.50 *μ*g/mL). Complementing the investigation of K^+^ channels, both incubation with the K_IR_ blocker (BaCl_2_; Figure 3(d)) and with the K_ATP_ blocker (GLB; Figure 3(e)) were unable to change the vasorelaxant activity of the extract, EC50 values of ~344.60 *μ*g/mL and ~360.80 *μ*g/mL, respectively. Together, these data suggest the participation of Ca^2+^-activated K^+^ channels (K_Ca_) and voltage-dependent K^+^ channels (K_V_) in the vasorelaxant activity induced by HEPB.

In addition to K^+^ channels, Ca^2+^ channels are intrinsically involved in the regulation of vascular tone, interacting with the contractile apparatus in the muscle cells of the vessels. The Ca^2+^ ion penetrates the cell cytoplasm through different channels; in the cardiovascular system, the most important channels are voltage-dependent (dependent on an electrical stimulus) and receptor-dependent (stimulated by agonists). The interaction of agonists (the classic mechanism of many vasoconstrictors, such as phenylephrine) with the G protein-coupled receptor activates a series of intracellular events that culminate in the formation of inositol triphosphate (IP_3_) and diacylglycerol (DAG). IP_3_ binds to its receptors in the sarcoplasmic reticulum, releasing the Ca^2+^ ion of this organelle to the cytosol. DAG activates the protein kinase C (PKC), which, in turn, phosphorylates L-type calcium channel-bound proteins, favoring the influx of extracellular Ca^2+^ into the intracellular environment. These two messengers produce the cytosolic elevation of Ca^2+^, causing the actin-myosin interaction and the contraction of vascular smooth muscle [27].

When activated, the *α* subunit of the G_s_ protein leads to the stimulation of adenylate cyclase, which leads to the formation of cyclic adenosine monophosphate (cAMP), from the breakdown of ATP. The cAMP activates protein kinase A, reducing intracellular Ca^2+^ concentration in vascular smooth muscle, with consequent vasodilation. However, the vascular relaxation promoted by forskolin is independent of the presence of functional endothelium [28], an effect that does not corroborate the present study's findings. Thus, it is suggested that the endothelium-dependent and relaxing effect promoted by HEPB does not depend on the generation of cGMP or cAMP. Based on this evidence, the involvement of forskolin in the vasorelaxant effect of *P. barbatus* extract is ruled out, reinforcing the absence of this compound in the chemical composition of the preparation or its presence in insignificant amounts.

**Figure 4:**
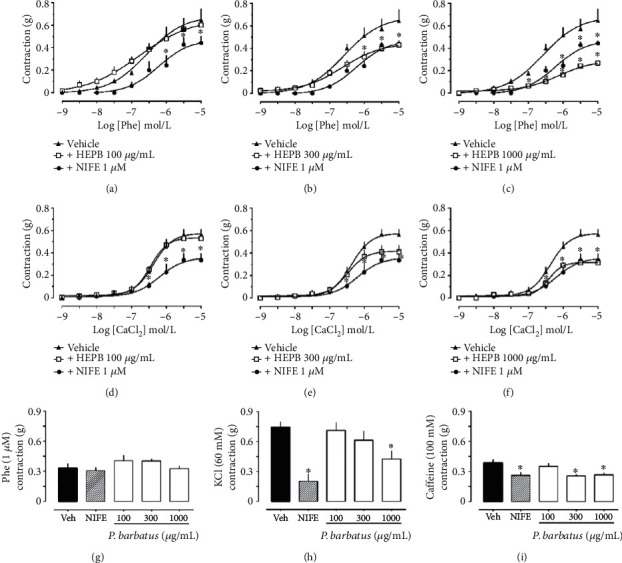
Role of Ca^2+^ channels in the relaxant effect induced by the hydroethanolic extract of *P. barbatus* (HEPB). Concentration-response curves to Phe in the presence or absence of HEPB: 100, 300, and 1000 *μ*g/mL (a, b, and c, respectively). Concentration-response curves to CaCl_2_ in the presence or absence of HEPB: 100, 300, and 1000 *μ*g/mL (d, e, and f, respectively). Contraction induced by Phe (g), KCl (h), and caffeine (i) in the presence of HEPB in calcium-free PSS. Nifedipine (NIFE) was used as a positive control. ^∗^*p* < 0.05 when compared to the vehicle group.

For a better understanding of the mechanisms responsible for the relaxing and modulating effect of vascular tone caused by HEPB, the previous addition of HEPB at concentrations of 300 and 1000 *μ*g/mL, but not 100 *μ*g/mL, was able to inhibit the contractile response to the cumulative addition of Phe, a selective agonist of the *α*1-adrenergic receptor (Figures 4(a)–4(c)). Nifedipine (NIFE), at a concentration of 1 *μ*M, was used as a positive control of the experiment, as it acts as a classical blocker of voltage-dependent calcium channels. Additionally, as shown in Figures 4(d)–4(f), it is possible to perceive that the contractile responses of the aortas of hypertensive rats exposed to the cumulative addition of CaCl_2_ in calcium-free depolarizing PSS were significantly reduced by the presence of HEPB at concentrations of 300 and 1000 *μ*g/mL, indicating that the influx of Ca^2+^ from the extracellular medium to the cytosolic medium is reduced in the presence of the extract. Thus, the data presented suggest that at least part of the vasorelaxant effect of the HEPB depends on the transmembrane channels for Ca^2+^, although so far, it is not possible to say whether directly and similar to the mechanism of action of the NIFE or indirect and related to another mechanism of action.

In complementarity, the channels for Ca^2+^ with localization in the sarcoplasmic reticulum membrane were also investigated. For this, we used 3 constricting agents (Phe, KCl, and caffeine) exposed to the aortic rings previously kept in a PSS free of Ca^2+^ to prevent its entry from the extracellular medium. Phe, as mentioned above, when binding to the *α*1-adrenergic receptor, culminates in the formation of IP3 and Ca^2+^ release. KCl, in turn, in high concentration in the extracellular medium, causes depolarization of vascular smooth muscle cells and the consequent opening of reticular Ca^2+^ channels, releasing them to the cytosol [29]. Caffeine interacts with receptors known as RyRs, due to its ability to bond with ryanodine, an alkaloid of plant origin. When caffeine binds to these receptors, there is a release of Ca^2+^ by the reticular reserves and the consequent stimulation of contraction of the muscular fibers of the vascular tissue [30]. As shown in Figure 4(g), the contraction induced by Phe in Ca^2+^-free PSS was not affected in the presence of any of the concentrations of the HEPB, suggesting that the receptors for IP3 located in the reticular membrane do not participate in the modulating actions of vascular tone promoted by the extract. On the other hand, in the presence of HEPB, aortic contraction induced with KCl (Figure 4(h)) or caffeine (Figure 4(i)) was significantly reduced compared to preparations exposed only to the vehicle, thus suggesting the participation of reticular receptors RyRs.

Even with the results obtained so far, we still need to investigate the relationship between the endothelium and the vascular smooth muscle in the relaxing effects evidenced by the administration of HEPB in an isolated aorta artery because the extract was exclusively endothelium-dependent in its vasodilatory actions. However, the main and predominant endothelium-derived mediator, nitric oxide, does not appear to be involved in the response. By analyzing the results in detail, we hypothesized that the key point of communication between the endothelium and vascular smooth muscle might be the K_CA_. In vasodilation, K^+^ itself may play a role in endothelium-derived hyperpolarization. When open, K_CA_ channels in the endothelium layer lead to the efflux of K^+^ to the cellular lumen or intercellular myoendothelial area. Consequently, endothelial cells are hyperpolarized and, as a result, transmit this hyperpolarization to adjacent cells (i.e., vascular smooth muscle cells (VSMC)) by direct electrical coupling throughout myoendothelial gap junctions. The K^+^ stored in the intercellular space can trigger K_IR_ and Na^+^/K^+^ ATPase activation in VSMC, generating hyperpolarization and voltage-gated Ca^2+^ channel blockade and culminating with the vasodilation [26, 27].

Before all these events, the entry of Ca^2+^ into the endothelial cell is fundamental for the activation of these channels. This occurs through a phosphorylation of the protein kinase G [31] or the attachment of other intermediate products to cysteine residues [32]. In fact, and corroborating with the findings of the present study, the activation of M_3_-type muscarinic receptors on endothelial cells stimulates this rise in cytosolic Ca^2+^ concentration. It is noteworthy that as Ca^2+^-sensitive K^+^ channels are implicated in endothelium-related reactions, activation of either endothelial or VSMC K_CA_ can avoid the incidence of endothelial dysfunction. Consequently, strategies that can activate or regulate the activity of these K^+^ channels may be of therapeutic significance [33], such as the preparation tested in this study.

## 4. Conclusion

In summary, the present results show, for the first time, that the hydroethanolic extract of *P. barbatus* leaves promotes a vasorelaxant effect in the thoracic aorta of hypertensive rats. The mechanism of action is exclusively dependent on the endothelium and mainly involves the participation of transmembrane channels for Ca^2+^.

## Figures and Tables

**Table 1:** Annotated compounds from the hydroethanolic extract of *P. barbatus* (HEPB).

Peak	RT (min)	MF	UV (nm)	Negative ion mode (*m/z*)		Positive ion mode (*m/z*)		Compound
				MS [M-H]^−^	MS/MS	MS [M+H]^+^	MS/MS	
1	1.1	C_6_H_8_O_7_	—	191.0202	—	215.0163^Na^	—	Citric acid
2	3.7	C_9_H_10_O_5_	281	197.0461	179, 151	221.0427^Na^	—	Syringic acid
3	7.7	C_14_H_20_O_7_	—	299.1146	—	323.1106^Na^	279, 246, 204, 189	Unknown
4	15.3	C_13_H_12_O_7_	299, 313	279.0529	163	303.0473^Na^	—	*p*-Coumaroyl malate
5	18.7	C_22_H_22_O_12_	270, 347	477.1053	314, 299, 285, 271, 255, 171	479.1189	317, 302, 191	*O*-Hexosyl *O*-methyl quercetin
6	18.9	C_21_H_18_O_12_	268, 348	461.0737	285, 257, 241, 175, 151	463.0872	287	*O*-Glucuronyl kaempferol
7	20.3	C_22_H_30_O_8_	—	421.1870	325, 308, 267, 195, 163	445.1845^Na^	385, 367, 309, 225	Unknown
8	21.2	C_18_H_16_O_8_	299, 328	359.0792	197, 179, 161	361.0916	—	Rosmarinic acid
9	23.9	C_16_H_12_O_7_	282, 342	315.0521	300, 271, 243, 202	317.0660	302, 274, 168	*O*-Methyl quercetin
10	24.4	C_19_H_32_O_10_	286, 334	421.2082	317	445.2057	333, 275, 229	Unknown
11	24.8	C_11_H_12_O_4_	299, 325	207.0671	179, 161	209.0809	163	Di-*O*-methyl caffeic acid
12	25.7	C_15_H_10_O_6_	268, 342	285.0410	257, 243, 225, 217, 183, 175	287.0557	269, 241, 153	Luteolin
13	27.6	C_24_H_32_O_9_	—	463.1985	—	465.2129	299, 281, 263, 239, 211, 160, 149	Cyclobutatusin
14	28.75	C_22_H_20_O_12_	286, 334	475.0884	299, 284	477.1053	301, 203, 167	*O*-Glucuronyl *O*-methyl scutellarein
15	29.0	C_15_H_10_O_5_	266, 336	269.0450	227, 202, 182, 171, 151	271.0597	243, 229, 201, 187, 169, 153	Apigenin
16	30.4	C_31_H_32_O_11_	330	579.1878	519, 475, 420, 395, 376, 323	581.2026	521, 203, 485, 411 393, 375, 367, 323, 309	Unknown
17	32.9	C_22_H_28_O_8_	280	419.1716	359, 331, 315, 272	421.1853	343, 325, 301, 273, 245, 219	Diterpenoid (coleon H)
18	35.4	C_22_H_32_O_5_	—	375.2180	313, 271, 259, 219	377.2313	299, 271, 217, 175, 161, 149	Unknown
19	37.1	C_27_H_46_O_9_	—	513.3078	317, 277, 209	515.3220	335, 261, 243, 187	Unknown

RT: retention time; MF: molecular formula. ^Na^[M+Na]^+^.

## Data Availability

The data used to support the findings of this study are available from the corresponding author upon request.

## Abbreviations

4-AP: 4-Aminopyridine
Ach: Acetylcholine
Atro: Atropine
cAMP: Cyclic adenosine monophosphate
cGMP: Cyclic guanosine monophosphate
DAG: Diacylglycerol
GLB: Glibenclamide
HEPB: Hydroethanolic extract of *P. barbatus*
Indo: Indomethacin
IP_3_: Inositol triphosphate
K_V_: Voltage-gated K^+^ channels
K_ATP_: ATP-sensitive K^+^ channels
K_IR_: Inward-rectifying K^+^ channels
K_Ca_: Ca^2+^-activated K^+^ channels
L-NAME: N*ω*-Nitro-L-arginine methyl ester hydrochloride
NIFE: Nifedipine
NO: Nitric oxide
NOS: Nitric oxide synthase
NSAID: Nonsteroidal anti-inflammatory drug
NTR: Normotensive rats
ODQ: 1H-[1,2,4]oxadiazolo[4,3-a]quinoxalin-1-one
Phe: Phenylephrine
Prop: Propranolol
PKC: Protein kinase C
PKA: Protein kinase A
sGC: Soluble guanylate cyclase
SHR: Spontaneously hypertensive rats
TEA: Tetraethylammonium
VSMC: Vascular smooth muscle cells.
